# Toxoplasma gondii Dysregulates Barrier Function and Mechanotransduction Signaling in Human Endothelial Cells

**DOI:** 10.1128/mSphere.00550-19

**Published:** 2020-01-29

**Authors:** Armond L. Franklin-Murray, Sharmila Mallya, Allen Jankeel, Suhas Sureshchandra, Ilhem Messaoudi, Melissa B. Lodoen

**Affiliations:** a Department of Molecular Biology & Biochemistry, University of California, Irvine, California, USA; b Institute for Immunology, University of California, Irvine, California, USA; University of California, Davis

**Keywords:** Hippo signaling, *Toxoplasma gondii*, VE-cadherin, actin, endothelial cell, mechanotransduction

## Abstract

Toxoplasma gondii can infect and replicate in vascular endothelial cells prior to entering host tissues. However, little is known about the molecular interactions at the parasite-endothelial cell interface. We demonstrate that T. gondii infection of primary human umbilical vein endothelial cells (HUVEC) altered cell morphology and dysregulated barrier function, increasing permeability to low-molecular-weight polymers. T. gondii disrupted vascular endothelial cadherin (VE-cadherin) and β-catenin localization to the cell periphery and reduced VE-cadherin protein expression. Notably, T. gondii infection led to reorganization of the host cytoskeleton by reducing filamentous actin (F-actin) stress fiber abundance under static and microfluidic shear stress conditions and by reducing planar cell polarity. RNA sequencing (RNA-Seq) comparing genome-wide transcriptional profiles of infected to uninfected endothelial cells revealed changes in gene expression associated with cell-cell adhesion, extracellular matrix reorganization, and cytokine-mediated signaling. In particular, genes downstream of Hippo signaling and the biomechanical sensor and transcriptional coactivator Yes-associated protein (YAP) were downregulated in infected endothelial cells. Interestingly, T. gondii infection activated Hippo signaling by increasing phosphorylation of LATS1, leading to cytoplasmic retention of YAP, and reducing YAP target gene expression. These findings suggest that T. gondii infection triggers Hippo signaling and YAP nuclear export, leading to an altered transcriptional profile of infected endothelial cells.

**IMPORTANCE** Toxoplasma gondii is a foodborne parasite that infects virtually all warm-blooded animals and can cause severe disease in individuals with compromised or weakened immune systems. During dissemination in its infected hosts, T. gondii breaches endothelial barriers to enter tissues and establish the chronic infections underlying the most severe manifestations of toxoplasmosis. The research presented here examines how T. gondii infection of primary human endothelial cells induces changes in cell morphology, barrier function, gene expression, and mechanotransduction signaling under static conditions and under the physiological conditions of shear stress found in the bloodstream. Understanding the molecular interactions occurring at the interface between endothelial cells and T. gondii may provide insights into processes linked to parasite dissemination and pathogenesis.

## Abstract (precis)

Toxoplasma gondii is a foodborne parasite that infects virtually all warm-blooded animals and can cause severe disease in individuals with compromised or weakened immune systems. During dissemination in its infected hosts, T. gondii breaches endothelial barriers to enter tissues and establish the chronic infections underlying the most severe manifestations of toxoplasmosis. The research presented here examines how T. gondii infection of primary human endothelial cells induces changes in cell morphology, barrier function, gene expression, and mechanotransduction signaling under static conditions and under the physiological conditions of shear stress found in the bloodstream. Understanding the molecular interactions occurring at the interface between endothelial cells and T. gondii may provide insights into processes linked to parasite dissemination and pathogenesis.

## INTRODUCTION

Endothelial cells line the blood vessels and play a pivotal role in maintaining homeostasis by controlling vascular permeability through specialized cell-to-cell junctions, such as the adherens and tight junctions (1, 2). Vascular endothelial cadherin (VE-cadherin), the major protein component of the adherens junction, connects the cytoskeleton of adjacent endothelial cells and stabilizes cell shape through interactions with actin (3). In this role, VE-cadherin is a major regulator of vascular permeability, and its dysregulation is linked to human pathologies (2).

VE-cadherin, actin, and associated binding proteins form a force-sensitive complex that enables rapid, global changes in endothelial cells in response to mechanical forces in the microenvironment (4). The Hippo pathway is highly conserved in mammals, and its signaling regulates cell growth, differentiation, and survival by inhibiting the transcriptional coactivator Yes-associated protein 1 (YAP) (5). Forces, such as the tension generated by shear stress, are detected by the adherens junctions and cytoskeleton and activate the Ste20-like protein kinases Mst1/2 (6). Large tumor suppressor kinase 1 (LATS1) is activated by Mst1/2 via phosphorylation at threonine residue 1079, resulting in YAP cytoplasmic retention and inhibition of YAP target gene expression (7, 8). Toll-like receptor (TLR) signaling has also been shown to activate the Hippo pathway in *Drosophila* (9). Interestingly, YAP is now appreciated as a key regulator of mammalian endothelial activation and inflammation (10), indicating that Hippo signaling is critical for endothelial cells to respond to vascular perturbations, such as coagulation, infection, or injury.

Toxoplasma gondii is an obligate intracellular parasite that infects an estimated one-third of the global population and causes significant morbidity and mortality in immunocompromised individuals (11). Humans are typically infected by consuming food or water contaminated with parasite cysts or through vertical transmission from mother to fetus. During dissemination in its host, T. gondii crosses formidable biological barriers, such as the blood-brain barrier (BBB), to exit the bloodstream and infect tissues where the parasite establishes a lifelong chronic infection (12). Current research suggests that T. gondii may leave the circulation to enter tissues inside motile immune cells that extravasate from the bloodstream or by directly infecting and lysing vascular endothelial cells (13). Indeed, T. gondii tachyzoites can adhere to and invade human vascular endothelium under shear stress conditions (14), and T. gondii can replicate in human retinal vascular endothelial cells (15). Recent evidence indicates that endothelial cells of the blood-brain barrier provide a replicative niche for T. gondii and facilitate parasite crossing of the BBB and entry into the central nervous system (CNS) (16). Despite a growing appreciation for the importance of endothelial infection in T. gondii pathogenesis, the molecular interactions occurring at this host-pathogen interface remain poorly defined.

In the present study, we investigated the morphological and functional consequences of T. gondii infection of primary human umbilical vein endothelial cells (HUVEC). We found that T. gondii infection dysregulated endothelial cell barrier function and remodeled the endothelial cell actin cytoskeleton. By conducting a global transcriptome analysis of infected endothelial cells, we identified gene expression changes associated with mechanotransduction and show that T. gondii infection activated Hippo signaling, as evidenced by LATS1 phosphorylation, and altered the subcellular localization of YAP, a protein that plays a critical role in sensing mechanical force and linking biomechanical stresses to gene expression changes in the cell.

## RESULTS

### T. gondii infection dysregulates endothelial cell barrier integrity and function.

**FIG 1:**
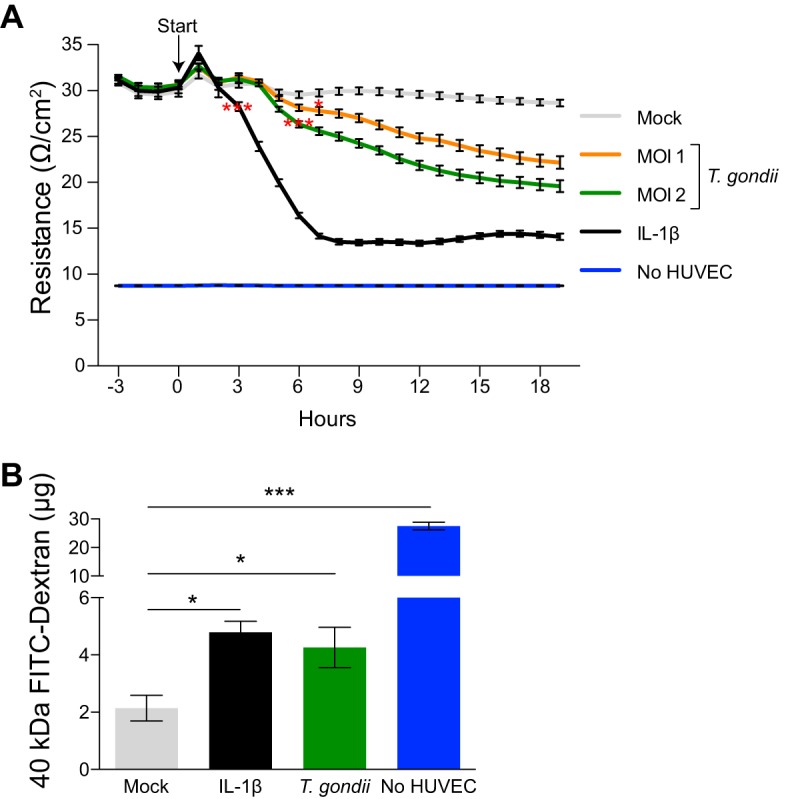
Effect of T. gondii infection on HUVEC barrier function. (A) Transendothelial electrical resistance (TEER) of mock-infected, T. gondii-infected, or IL-1β-treated (0.5 ng/ml) HUVEC monolayers is shown. HUVEC were cultured to confluence for 3 days. T. gondii or IL-1β was added at time zero (start), and barrier integrity was measured at 15-min intervals for 18 h by ECIS. Data reflect combined results from three independent experiments and are presented as means ± standard errors of the means (SEM) (error bars). Values that are significantly different by two-way ANOVA with a Bonferroni posttest correction are indicated by asterisks as follows: *, *P* < 0.05; ***, *P* < 0.001. (B) Permeability of mock-infected, T. gondii-infected, or IL-1β-treated (0.5 ng/ml) HUVEC monolayers, as measured by transendothelial flux of 40-kDa FITC-dextran at 18 h. Flux is presented as means ± SEM. Data reflect combined results from three independent experiments. Values that are significantly different by one-way ANOVA with a Tukey posttest are indicated by bars and asterisks as follows: *, *P* < 0.05; ***, *P* < 0.001.

**FIG 2:**
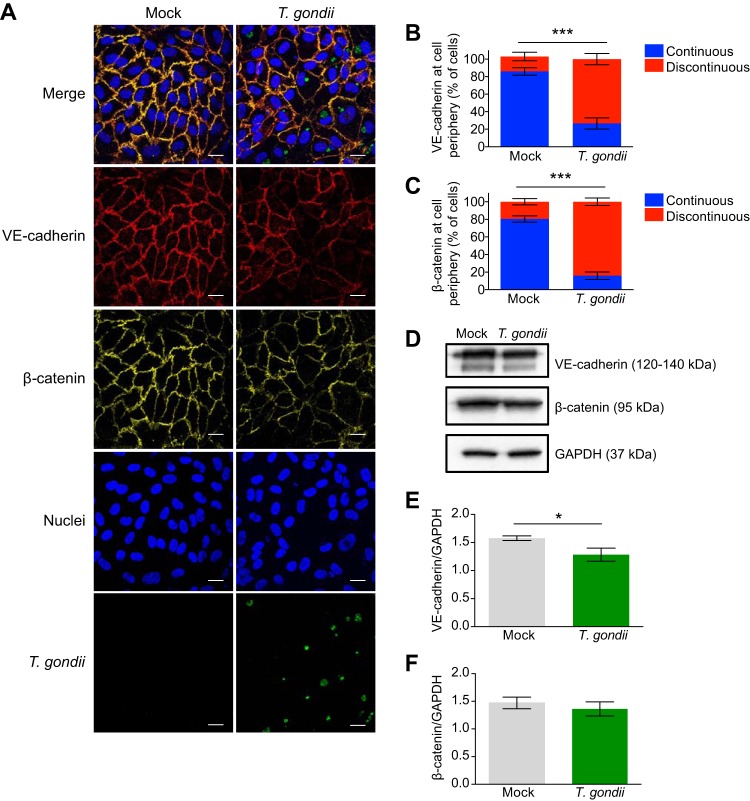
Adherens junction protein expression in T. gondii-infected HUVEC. (A) HUVEC were cultured to confluence on fibronectin-coated glass coverslips for 3 days and then either mock infected with fresh media or infected with T. gondii. At 18 hpi, the cells were fixed, permeabilized, and stained with antibodies specific to VE-cadherin and β-catenin and counterstained with DAPI. Bars, 20 μm. (B and C) The continuity of VE-cadherin and β-catenin signal at the cell periphery was quantified by examining immunofluorescent signal of VE-cadherin at the cell periphery and defining gaps of ≥2.5 μm as discontinuous signal. Data are presented as the means ± SEM from at least three independent experiments. Student’s *t* test was performed (***, *P* < 0.001). (D) HUVEC were cultured to confluence for 3 days and either mock infected with fresh media or infected with T. gondii. At 18 hpi, cells were lysed and analyzed by Western blotting using antibodies for total VE-cadherin, β-catenin, and glyceraldehyde-3-phosphate dehydrogenase (GAPDH). Representative blots from five independent experiments are shown. (E and F) Densitometry was performed on Western blots of VE-cadherin and β-catenin by normalizing band intensities to those of GAPDH. Data are presented as the means ± SEM. Combined data from five independent experiments are shown. Student’s *t* test was performed (*, *P* < 0.05).

**FIG 3:**
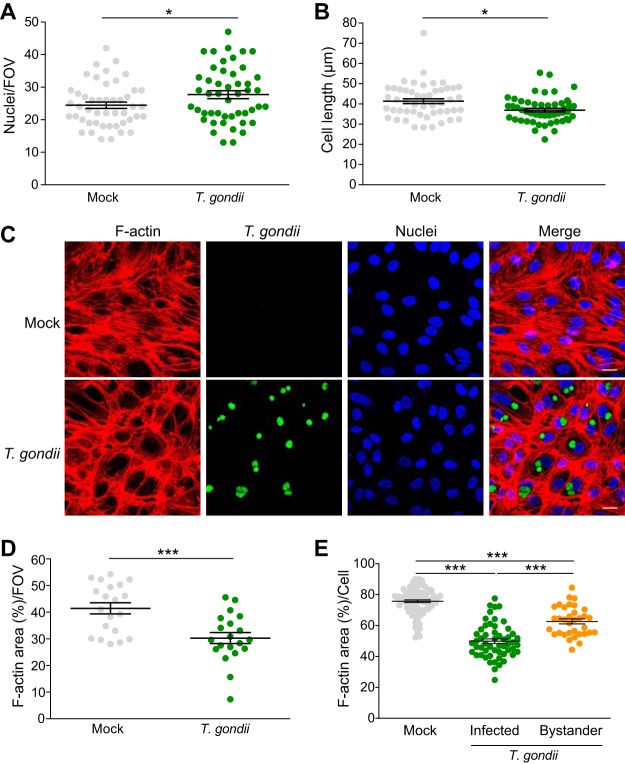
Cell morphology and stress fiber abundance in HUVEC during T. gondii infection. HUVEC were cultured to confluence on fibronectin-coated glass coverslips for 3 days and then either mock infected with fresh media or infected with T. gondii. Eighteen hours later, the cells were fixed, permeabilized, and stained to detect nuclei, VE-cadherin, or F-actin for confocal microscopy. (A) The number of nuclei per 63× field of view (FOV) was quantified. Each symbol represents one FOV. (B) The maximal cell length of individual HUVEC in each condition was determined. Each symbol represents the value for an individual cell. The means (horizontal lines) ± SEM (error bars) from 3 to 10 independent experiments are shown. Values that are significantly different (*P* < 0.05) by Student’s *t* test are indicated by a bar and asterisk. (C) Representative images of F-actin (as detected by phalloidin staining) and DAPI in mock-infected and T. gondii-infected HUVEC are shown. Bars, 20 μm. (D and E) Percent area of F-actin per 63× FOV (D) or F-actin area per cell (E) under each condition are shown. The means ± SEM (error bars) from four independent experiments are shown. Statistical significance was analyzed by Student’s *t* test for panels A, B, and D and by one-way ANOVA with a Tukey posttest correction for panel E and is indicated by asterisks as follows: *, *P* < 0.05, ***, *P* < 0.001.

**FIG 4:**
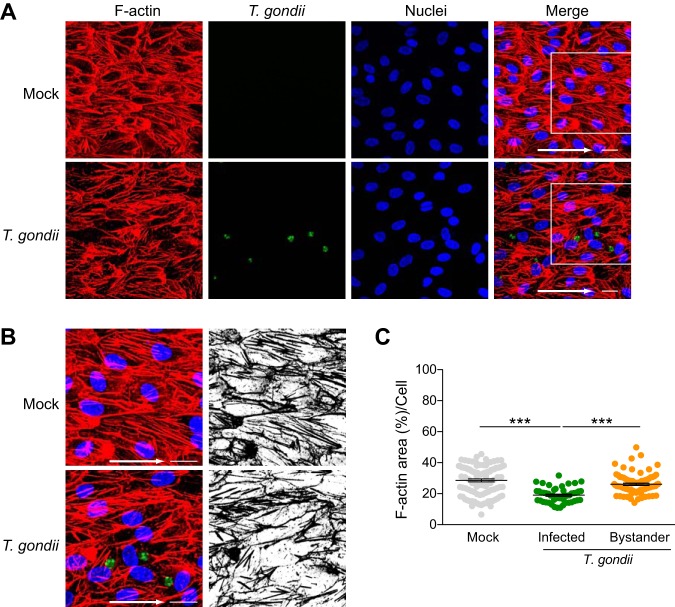
Stress fiber abundance in HUVEC exposed to shear stress during T. gondii infection. HUVEC were cultured to confluence on ibiTreat 0.4-luer ibidi microchannels under 5.5-dyne/cm^2^ shear stress for 3 days and either mock infected with fresh media or infected with T. gondii. At 18 hpi, the samples were fixed, permeabilized, and stained with phalloidin to detect F-actin. (A) Fluorescence microscopy of F-actin, GFP-expressing T. gondii, and nuclei under 5.5-dyne/cm^2^ shear stress in mock-infected and T. gondii-infected cells at 18 hpi is shown. The arrows in the merged images indicate the direction of flow. Bars = 20 μm. (B) Magnified insets of the white squares shown in the merged images of panel A, with F-actin (red), GFP-expressing T. gondii (green), and nuclei (blue) in mock-infected and T. gondii-infected HUVEC exposed to 5.5-dyne/cm^2^ shear stress. Black and white images show F-actin only. (C) Percent area of F-actin on a per cell basis is shown. The means ± SEM (error bars) from four independent experiments are shown. One-way ANOVA with a Tukey posttest correction was performed (***, *P* < 0.001).

T. gondii can infect endothelial cells to exit the bloodstream and enter host tissues, such as the lung and CNS (16). To examine the effect of infection on vascular endothelial barrier integrity, electrical cell-substrate impedance sensing (ECIS) assays were used. HUVEC were seeded into fibronectin-coated wells in an ECIS plate and cultured to confluence for 72 h (see Fig. S1 in the supplemental material). The cells were then mock infected with fresh media, infected with green fluorescent protein (GFP)-expressing type II (*Prugniaud* strain) T. gondii at a multiplicity of infection (MOI) of 1 or 2, or treated with interleukin-1beta (IL-1β) as a positive control to induce barrier permeability. Impedance measurements were made prior to treatment and continuously every 15 min for at least 18 h (Fig. 1A). Mock-infected HUVEC showed no significant changes in transendothelial electrical resistance (TEER) throughout the time frame of analysis, whereas HUVEC treated with IL-1β exhibited a reduction in TEER beginning at 3 h, which progressed to 6 h. Interestingly, T. gondii infection of HUVEC reduced TEER as early as 6 hpi. The reduction in TEER persisted over time, and this effect was dose dependent, as TEER levels decreased with increasing parasite MOI. Decreased TEER measurements in infected cells did not appear to be the result of cell lysis, as visual inspection by microscopy confirmed intact monolayers at 18 hpi with the dose and strain of T. gondii used in all experiments (see Fig. 2 to 4). These data suggest that T. gondii infection of host endothelial cells reduced barrier integrity within 6 h after infection.

10.1128/mSphere.00550-19.1 FIG S1 HUVEC barrier integrity during the establishment of monolayers. Subconfluent HUVEC were seeded on fibronectin in an ECIS plate, and the TEER values of HUVEC or media alone (No HUVEC) were measured every 15 min for 72 h, during which the cells established a confluent monolayer. HUVEC were fed with fresh media at 24 h and 48 h. Data points for each time point show the means ± SEM from 12 wells per condition. One representative experiment of three independent experiments is shown. Download FIG S1, PDF file, 0.1 MB. Copyright © 2020 Franklin-Murray et al. 2020 Franklin-Murray et al. This content is distributed under the terms of the Creative Commons Attribution 4.0 International license.

To further test barrier function in endothelial cells, we examined whether T. gondii infection of endothelial monolayers altered permeability to a low-molecular-weight polymer in a transwell assay. HUVEC were seeded onto fibronectin-coated 0.4-μm porous membrane inserts in the apical chamber and cultured to confluence for 72 h. Confluent monolayers were mock infected, infected with T. gondii (MOI of 2), or treated with IL-1β as a positive control for 18 h. For the final 30 min of culture, 40-kDa fluorescein isothiocyanate (FITC)-dextran was added to the apical chamber, and the contents of the basolateral chamber were collected to evaluate endothelial barrier permeability (Fig. 1B). As expected, IL-1β treatment increased the amount of FITC-dextran passing through the HUVEC monolayer compared to mock-infected cells. T. gondii infection also increased monolayer permeability 1.99-fold at 18 h postinfection (hpi) compared to mock-infected cells. These results indicated that endothelial cell barrier function decreased during T. gondii infection of HUVEC.

### T. gondii infection leads to decreased VE-cadherin localization to the cell periphery.

Adherens junctions are critical for the formation and maintenance of endothelial barriers, and VE-cadherin is the main determinant of the integrity of these junctions (2, 17, 18). To investigate whether the observed changes in barrier permeability and function coincided with altered levels of VE-cadherin in endothelial cells following infection, HUVEC were cultured to confluence for 72 h on fibronectin. They were then mock infected with fresh media or infected with GFP-expressing T. gondii. At 18 hpi, the monolayers were analyzed for VE-cadherin signal by immunofluorescence microscopy (Fig. 2A). Mock-infected monolayers showed a zipper-like VE-cadherin staining pattern between adjacent cells that is characteristic of mature adherens junctions. Continuity of VE-cadherin staining was quantified on an individual cell basis by examining immunofluorescent signal of VE-cadherin at the cell periphery and defining gaps of ≥2.5 μm as discontinuous signal (Fig. S2). Continuous VE-cadherin staining was observed in 83% of mock-infected HUVEC over multiple experiments (Fig. 2B). In contrast, continuous VE-cadherin was observed in only 27% T. gondii-infected HUVEC (Fig. 2B), as infection led to a marked reduction in VE-cadherin at the cell periphery, gaps in VE-cadherin staining, and partial loss of VE-cadherin signal in some infected cells (Fig. 2A). By examining total VE-cadherin protein levels from endothelial cell lysates, an 18.7% reduction in VE-cadherin protein levels was observed in infected cells at 18 hpi (Fig. 2D and E).

10.1128/mSphere.00550-19.2 FIG S2 VE-cadherin continuity/discontinuity analysis. HUVEC were cultured to confluence for 72 h on fibronectin-coated glass coverslips and either mock infected with fresh media or infected with GFP-expressing T. gondii. After 18 h, the samples were fixed, permeabilized, and stained with antibodies specific to VE-cadherin and counterstained with DAPI. Gaps in signal of  ≥2.5 μm were considered to be indicative of discontinuous junctions and are indicated by red arrows. Images reflect data from one of four independent experiments. Bars, 20 μm. Download FIG S2, EPS file, 3.4 MB. Copyright © 2020 Franklin-Murray et al. 2020 Franklin-Murray et al. This content is distributed under the terms of the Creative Commons Attribution 4.0 International license.

The cytoplasmic tail of VE-cadherin interacts with β-catenin to promote stability of endothelial junctions (19). Therefore, we also examined whether T. gondii infection dysregulated β-catenin localization during infection. Continuous β-catenin staining was observed in 80% of mock-infected HUVEC compared to 16% of infected HUVEC (Fig. 2C). We observed that β-catenin localization to the cell periphery appeared dysregulated in infected HUVEC in a manner similar to VE-cadherin localization (Fig. 2A). However, Western blotting did not reveal changes in total protein levels of β-catenin at 18 hpi (Fig. 2D and F).

### T. gondii infection alters cell morphology and F-actin stress fiber abundance.

Adherens junctions are linked to the actin cytoskeleton to maintain stability and cell shape (2, 19). In addition to detecting changes in VE-cadherin at the cell periphery, we also observed changes in the morphology of T. gondii-infected endothelial cells. At 18 hpi, more nuclei were observed per field of view (FOV) (Fig. 3A), and the maximal cell length was decreased by 11% in T. gondii*-*infected HUVEC compared to mock-infected HUVEC (Fig. 3B). To determine whether these subtle changes in morphology were associated with cytoskeletal rearrangements, we investigated whether infection altered the actin cytoskeleton by staining the cells with fluorescently conjugated phalloidin and examining the cells by confocal microscopy (Fig. 3C). Quantification of the filamentous actin (F-actin) area per FOV revealed that F-actin abundance was significantly reduced in infected monolayers (Fig. 3D). Notably, the cytoplasmic network of F-actin stress fibers appeared to be rearranged in infected HUVEC by 18 hpi. By examining F-actin on an individual cell level, we determined that T. gondii infection reduced cytoplasmic stress fiber abundance by 34.15% in directly infected cells and by 17.3% in bystander cells (Fig. 3E). The effect on F-actin was observed in cells with both large and small vacuoles (Fig. 3C and Fig. S3). These data suggest that T. gondii modulates actin cytoskeleton organization in HUVEC during infection.

10.1128/mSphere.00550-19.3 FIG S3 F-actin signal in T. gondii-infected HUVEC. HUVEC were cultured to confluence for 72 h on fibronectin-coated glass coverslips and either mock infected with fresh media or infected with T. gondii. After 18 h, the samples were fixed, permeabilized, and stained to detect F-actin using phalloidin and nuclei using DAPI. Bars, 20 μm. (A) Fluorescence microscopy showing cells with small vacuoles and F-actin disruption at 18 hpi. (B) Fluorescence microscopy showing larger vacuoles and F-actin disruption at 18 hpi. Black and white image shows F-actin signal only. For panels A and B, the images reflect data from at least three independent experiments. (C) Percent area of F-actin on a per cell basis are shown and binned by the total number of parasites counted in the infected cells at 18 hpi. Data reflect combined results from at least three independent experiments. One-way ANOVA with a Tukey posttest was performed (n.s., not significant). Download FIG S3, EPS file, 10.0 MB. Copyright © 2020 Franklin-Murray et al. 2020 Franklin-Murray et al. This content is distributed under the terms of the Creative Commons Attribution 4.0 International license.

### Planar cell polarity is disrupted in T. gondii-infected endothelial cells.

In the bloodstream, the mechanical forces caused by fluidic shear stress alter cell morphology and the arrangement of the cytoskeleton (20–23). To further investigate the effect of T. gondii infection on the endothelial cell actin cytoskeleton, we determined whether the changes in F-actin detected in static cultures were also observed under more physiological shear stress conditions. HUVEC were seeded in ibidi microfluidic chambers and cultured under unidirectional continuous flow for 72 h at 5.5-dyne/cm^2^ shear stress to simulate conditions in postcapillary venules (24). As previously reported (22), HUVEC adopted a polarized morphology under flow conditions, in which the cells aligned in the direction of flow (Fig. 4A). The cells were then mock infected or infected with GFP-expressing T. gondii. After 18 h, the monolayers were stained with phalloidin to detect F-actin. Consistent with the findings in static conditions, T. gondii also disrupted cytoplasmic F-actin organization in infected HUVEC under shear stress conditions (Fig. 4A and B). Cytoplasmic stress fiber abundance was reduced by 35.7% in directly infected cells and by 8.5% in bystander cells compared to mock-infected HUVEC (Fig. 4C).

**FIG 5:**
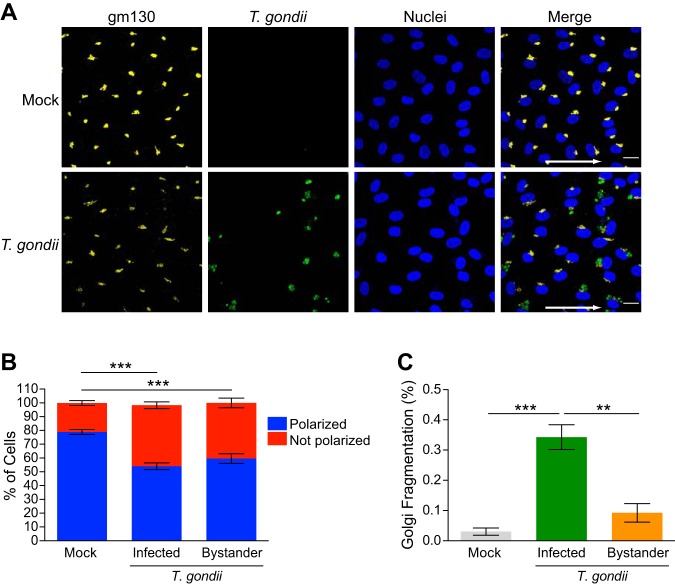
Effect of T. gondii infection on HUVEC planar cell polarity. HUVEC were cultured to confluence on ibiTreat 0.4-luer ibidi microchannels under 5.5-dyne/cm^2^ shear stress for 3 days and either mock infected with fresh media or infected with T. gondii. At 18 hpi, the samples were fixed, permeabilized, and stained with anti-gm130 to detect the Golgi complex. (A) Fluorescence microscopy of gm130, GFP-expressing T. gondii, and nuclei at 5.5-dyne/cm^2^ shear stress in mock-infected and T. gondii-infected cells at 18 hpi is shown. The arrows in the merged images indicate the direction of flow. Bars = 20 μm. (B) Planar cell polarity of mock-infected and T. gondii-infected HUVEC at 18 hpi is shown. Combined data from three independent experiments are presented as the means ± SEM. One-way ANOVA with a Tukey posttest correction was performed (***, *P* < 0.001). (C) The percentage of cells exhibiting Golgi fragmentation was quantified in the mock-infected and T. gondii-infected cultures at 18 hpi. Directly infected cells and bystander cells in the infected cultures are shown. Data are presented as means ± SEM from three independent experiments. One-way ANOVA with a Tukey posttest correction was performed (***, *P* < 0.001; **, *P* < 0.01).

We next determined whether the observed disruption in F-actin impacted HUVEC planar cell polarity. The position of the Golgi apparatus relative to the nucleus provides an indication of planar cell polarity (25). When the Golgi position was evaluated in mock-infected monolayers by staining for gm130 as a marker of the Golgi apparatus, 79% of the cells displayed Golgi localization consistent with a polarized phenotype (Fig. 5A and B). In contrast, there was reduced planar cell polarity in T. gondii-infected cultures, in which 54% and 59% of the cells were polarized in directly infected and bystander cells, respectively (Fig. 5A and B). In addition, it was noted that T. gondii caused Golgi fragmentation under shear stress conditions (Fig. 5A), as previously reported for static culture conditions (26). Interestingly, Golgi fragmentation was significantly increased in directly infected HUVEC compared to both mock-infected or bystander cells (Fig. 5C). Collectively, these data demonstrate that T. gondii infection of endothelial cells led to altered F-actin distribution and abundance, as well as reduced planar cell polarity in cells cultured under microfluidic shear stress conditions.

### T. gondii infection induces gene expression changes in endothelial cells.

**FIG 6:**
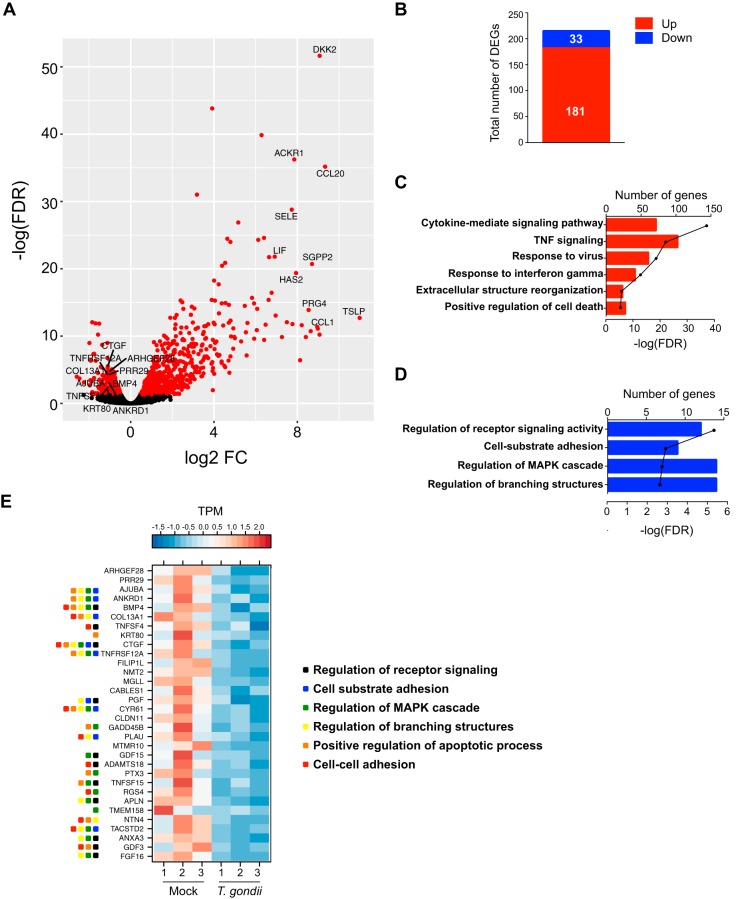
RNA-Seq in mock-infected or T. gondii-infected HUVEC at 18 hpi. HUVEC were mock infected with fresh media or infected with T. gondii at an MOI of 2 for 18 h, and RNA-Seq was performed on samples from three independent experiments. (A) Volcano plot depicting total gene expression changes in HUVEC at 18 hpi with red representing significantly differentially expressed genes (DEGs) (FDR < 0.05) and black representing nonsignificantly changed genes (FDR ≥ 0.05). The top 10 upregulated and downregulated DEGs with RPKM of >5 are labeled. FC, fold change. (B) Total number of DEGs (fold change of >2, FDR < 0.05, RPKM > 5) that were up- or downregulated by infection. (C and D) Bar graphs show Gene Ontology (GO) terms for gene sets with increased (C) or decreased (D) transcript abundance at 18 hpi. The number of genes within each GO term is shown, and the black line represents the −log (FDR-adjusted *P* value) for each GO term. TNF, tumor necrosis factor. (E) Heatmaps represent expression of the 33 downregulated DEGs at 18 hpi. Normalized TPM (transcripts per million) values are shown. The range of colors is based on scaled and centered TPM values of the entire set of genes (red represents high expression, and blue represents low expression). For genes that mapped to multiple GO pathways, the pathways are indicated by colored squares.

To further investigate T. gondii-induced changes in cell morphology and barrier function, we examined changes in the gene expression profiles of mock-infected and T. gondii-infected HUVEC at 18 hpi using RNA sequencing (RNA-Seq). Infection resulted in a greater number of upregulated genes than downregulated genes (Fig. 6A). Among the most abundant transcripts at 18 hpi were *CCL1* (chemokine ligand 1), *CCL20* (chemokine ligand 20), *HAS2* (hyaluronan synthase 2), and *SELE* (selectin E), which are markers of endothelial activation in response to inflammatory stimuli (Fig. 6A). We identified 214 protein-coding genes as differentially expressed between mock-infected and T. gondii-infected HUVEC that met the following criteria: fold change > 2, false-discovery rate (FDR) < 0.05, and reads per kilobase per million (RPKM) > 5. Among these differentially expressed genes (DEGs), 181 were increased in transcript abundance and 33 were decreased in transcript abundance (Fig. 6B). Functional enrichment using Metascape revealed that gene sets with increased transcript abundance mapped to Gene Ontology (GO) pathways associated with cytokine-mediated signaling, extracellular structure reorganization, and regulation of cell death (Fig. 6C). Many of the genes in these pathways are involved in inflammatory responses and signaling, as has been previously reported during T. gondii infection of other cell types (27–29). On the other hand, gene sets with decreased transcript abundance during infection mapped to GO terms related to the regulation of receptor signaling, cell adhesion, and regulation of mitogen-activated protein kinase (MAPK) signaling and branching structures (Fig. 6D). Interestingly, T. gondii infection reduced transcript abundance of *ANKRD1* (ankyrin repeat domain 1), *CTGF* (connective tissue growth factor), and *CYR61* (cysteine-rich angiogenic inducer 61), three genes that are associated with mechanotransduction signaling pathways linked to changes in the actin cytoskeleton (30–32) (Fig. 6E).

### YAP activity is altered in T. gondii-infected endothelial cells.

**FIG 7:**
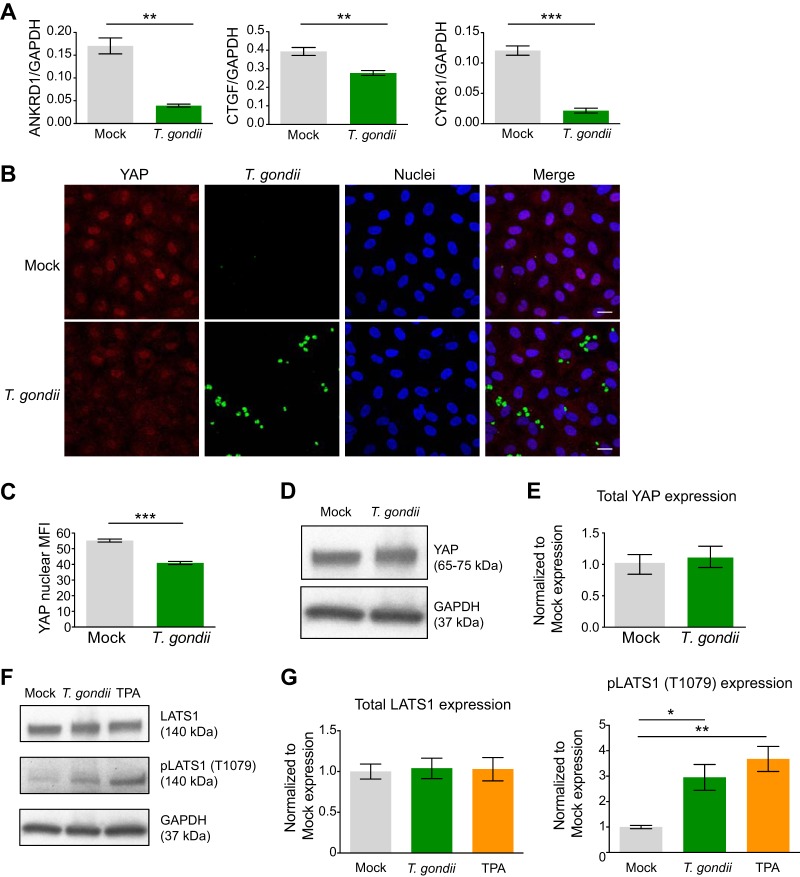
Activation of Hippo signaling in HUVEC during T. gondii infection. HUVEC were mock infected with fresh media or infected with T. gondii at an MOI of 2 for 18 h. (A) qPCR was performed with specific primers for ANKRD1, CTGF, CYR61, or GAPDH. The transcript levels relative to those of GAPDH from one representative experiment out of a total of three experiments are shown. Student’s *t* test was performed (**, *P* < 0.01; ***, *P* < 0.001). (B) HUVEC were mock infected or infected with GFP-expressing T. gondii, and 18 h later, the cells were fixed, permeabilized, and stained with antibody to detect YAP and counterstained with DAPI. Bars, 20 μm. (C) The mean fluorescence intensity (MFI) of nuclear YAP in mock-infected and T. gondii-infected cells at 18 hpi is shown. Combined data from three independent experiments are shown and presented as the means ± SEM. Student’s *t* test was performed (***, *P* < 0.001). (D and F) HUVEC were cultured to confluence and either mock infected, infected with T. gondii, or treated with TPA. Eighteen hours later, the cells were lysed and analyzed by Western blotting using antibodies for total YAP, total LATS1, phospho-LATS1 (Thr1079), or GAPDH. Representative blots from three independent experiments are shown. (E and G) Densitometry was performed on Western blots of total YAP, total LATS1 protein, and phospho-LATS1 (Thr1079) by normalizing band intensities to those of GAPDH. Data are presented as means ± SEM. Combined data from five independent experiments are shown. One-way ANOVA with a Tukey posttest correction was performed (*, *P* < 0.05).

Mechanotransduction plays an integral role in the ability of cells to sense the external environment and respond to mechanical force to regulate cell proliferation and shape (30). In endothelial cells, the actin cytoskeleton forms a mechanosensitive complex through protein interactions with the adherens junctions to detect changes in extracellular matrix (ECM) stiffness, cell-cell contacts, and shear stress (4). Signals from the plasma membrane and changes in F-actin stress fiber organization modulate the activity of the transcriptional coactivator Yes-associated protein (YAP), a key downstream effector of canonical Hippo signaling. In response to mechanical stress or disruptions in cell adhesion, Hippo signaling induces Mst1/2-mediated phosphorylation of LATS1, leading to YAP nuclear export and repression of YAP target gene transcription (33). The expression of YAP target genes *ANKRD1*, *CTGF*, and *CYR61* was reduced in infected HUVEC compared to mock-infected endothelial cells (Fig. 6E). Using the same mRNA samples as in the RNA-Seq experiments, we validated the RNA-Seq results for these genes using quantitative PCR (qPCR) with primers specific for *ANKRD1*, *CTGF*, and *CYR61* and confirmed reduced mRNA transcript abundance for each of these genes in T. gondii-infected endothelial cells compared to mock-infected endothelial cells (Fig. 7A).

To investigate whether T. gondii infection affected YAP subcellular localization, we performed immunofluorescence microscopy for YAP in HUVEC during infection. Mock-infected HUVEC expressed strong nuclear YAP signal and weak cytoplasmic signal, consistent with these cells being at confluence on a relatively stiff surface of fibronectin on glass (30). Interestingly, YAP nuclear localization appeared reduced in T. gondii-infected HUVEC (Fig. 7B). Quantification of nuclear YAP signal revealed a 22% reduction in mean fluorescence intensity (MFI) at 18 hpi (Fig. 7C). We did not detect any statistically significant changes in YAP mRNA by RNA-Seq or YAP protein expression by Western blot analysis (Fig. 7D and E), suggesting that YAP was most likely regulated at the level of its subcellular localization in infected HUVEC.

We next determined whether T. gondii infection induced the Hippo signaling pathway in endothelial cells by examining phosphorylation of the kinase LATS1, which is upstream of the Hippo effector YAP. Western blotting revealed no changes in total LATS1 protein expression, but we observed increased phosphorylation of LATS1 at Thr1079 in T. gondii*-*infected cells at 18 hpi compared to mock-infected cells (Fig. 7F and G). 12-*O*-Tetradecanoylphorbol-13-acetate (TPA) was used as a positive control to induce phospho-LATS1 (Fig. 7F and G). These findings support a model whereby T. gondii infection modulates pathways relevant to mechanical stress in endothelial cells through the Hippo signaling pathway, leading to phosphorylation of LATS1 and cytoplasmic retention of YAP, thereby reducing YAP nuclear activity and target gene expression.

## DISCUSSION

Actin is the most abundant, highly conserved protein in mammalian cells and constitutes about 10% of total protein in endothelial cells (34, 35). The dynamic polymerization and depolymerization of actin filaments are important for organelle position, intracellular trafficking, ECM sensing, and cell shape (36–38). Filamentous actin interacts with catenins to anchor interendothelial cell junctions and stabilize endothelial barrier integrity (37). Actin, VE-cadherin, and associated binding proteins form a mechanosensing complex in endothelial cells (4). As a result, changes in the external environment, such as mechanical forces, are transduced through the actin cytoskeleton into cellular responses (33).

As the cells that line the blood vessel walls, vascular endothelial cells are exposed to the shear stress of rapidly flowing blood. Shear stress induces profound morphological and functional adaptations via mechanotransduction signaling in vascular endothelial cells (20–22). Planar cell polarity, which is the alignment of cells in a tissue plane, is induced by shear stress (23). This polarity provides directionality to intracellular trafficking, and if dysregulated, can contribute to a variety of developmental diseases, as well as vascular pathologies, such as polycystic kidney cancer (23, 39, 40). In examining the extent to which T. gondii infection altered the actin cytoskeleton of endothelial cells under continuous shear stress conditions, we found that infection disrupted planar cell polarity and led to Golgi fragmentation, indicating potential dysregulation of vesicle trafficking. T. gondii is reported to fragment the host cell Golgi apparatus and reposition the functional ministacks around its parasitophorous vacuole to modulate vesicular trafficking and scavenge sphingolipids in nonendothelial cell types (26). In endothelial cells, vesicular transport is important for VE-cadherin localization to the plasma membrane and adherens junction stability (41). As a result, T. gondii disruption of vesicular transport may disrupt VE-cadherin trafficking to cell-cell junctions in infected HUVEC, thereby contributing to barrier dysregulation and increased paracellular permeability. This may explain the modest reduction in VE-cadherin total protein levels observed during infection, despite the dramatic difference in localization at the plasma membrane that was observed by confocal microscopy. Interestingly, a recent study by Ross et al. demonstrated that extracellular parasites can transmigrate across brain endothelial cells without perturbing barrier integrity (42). T. gondii decreased phosphorylation of focal adhesion kinase (FAK), a regulator of endothelial tight junctions, to transiently destabilize endothelial barriers without lysing the host cell (42). This adds to growing evidence that live infection with T. gondii affects FAK regulation, perhaps aiding the parasite in dissemination (43, 44).

A variety of obligate intracellular pathogens modulate endothelial cell biology and function during infection (45). Gram-negative bacteria, such as pathogenic *Rickettsia* species and Chlamydia trachomatis alter cell morphology and function in infected endothelial cells to facilitate intracellular motility, cell-to-cell dissemination, and nutrient acquisition by intercepting vesicular transport from the host cell Golgi apparatus (46–48). Both *Rickettsia* species and C. trachomatis disrupt adherens junctions and increase vascular permeability during infection by inducing VE-cadherin phosphorylation (49, 50). Furthermore, intracellular replication of these pathogens within endothelial cells leads to an activated, proinflammatory endothelial phenotype, which may also contribute to increased barrier permeability, since inflammatory cytokine signaling is known to both upregulate adhesion molecules and reduce barrier integrity.

T. gondii infection results in a proinflammatory gene expression profile in several cell types (27–29, 51). Consistent with these previous reports, our RNA-Seq data confirm that T. gondii induces proinflammatory genes in human endothelial cells during infection. Functional enrichment revealed upregulated DEGs associated with the immune response and immune cell adhesion, such as *ICAM-1* (intercellular adhesion molecule 1), *VCAM-1* (vascular cell adhesion molecule 1), *CXCL3* (chemokine ligand 3), and *SELE* (selectin E). By analyzing the F-actin area in directly infected cells compared to bystander cells in the same culture, we observed that cells that were directly infected by T. gondii had the greatest reduction in F-actin area compared to mock-infected cells; however, the uninfected bystander cells had an intermediate phenotype, suggesting that soluble inflammatory factors acting in a paracrine manner may contribute to this bystander effect, which was more apparent under static culture conditions than under conditions of fluidic shear stress. Although HUVEC barrier integrity was reduced in response to IL-1β treatment, as demonstrated by TEER assays, the RNA-Seq data demonstrated that T. gondii infection of HUVEC did not induce IL-1β transcripts, suggesting that other inflammatory mediators may contribute to the bystander effect in the infected HUVEC culture.

T. gondii is also reported to induce quiescent cells into S phase, thereby facilitating host cell cycle progression (52–54). Interestingly, we detected increased expression of genes related to cell proliferation, such as *G0S2* (G_0_/G_1_ switch 2) and *EGR1* (early growth response 1), which may help to explain the increased number of nuclei per FOV detected in the infected-cell cultures.

YAP plays a central role in mechanotransduction by enabling cells to detect perturbations in actin cytoskeleton organization caused by mechanical forces and transducing these signals into changes in gene transcription (30, 31, 33). Our RNA-Seq analysis revealed a subset of downregulated DEGs that were associated with receptor signaling activity, cell-substrate adhesion, and regulation of cellular proliferation. Among these DEGs, we observed reduced mRNA transcripts for three YAP target genes: *ANKRD1*, *CTGF*, and *CYR61*. *ANKRD1* is a transcriptional cofactor that negatively regulates matrix metalloproteinase transactivation and plays a vital role in wound healing (55). CTGF and CYR61 are matricellular proteins of the CCN (*c*onnective tissue growth factor, *c*ysteine-rich protein, and *n*ephroblastoma overexpressed gene) family that are secreted into the ECM to regulate cell adhesion, cell proliferation, and vascular remodeling (56, 57). YAP lacks DNA-binding activity and must interact with a DNA-binding protein to enter the nucleus and modulate expression of its target genes (58). YAP interacts with TEAD (TEA/ATTS domain) transcription factors to drive expression of *ANKRD1*, *CTGF*, *CYR61*, and other genes important for cell proliferation. Alterations in the activity of YAP and expression of YAP target genes were correlated with upstream phosphorylation of LATS1 at Thr1079, suggesting that T. gondii infection of endothelial cells activates a Hippo signaling cascade, likely due to changes in mechanical stress on the host cell.

YAP activity depends on its subcellular localization, which can be modulated through phosphorylation at serine residue 127 via Hippo signaling and subsequent interactions with 14-3-3 proteins, which anchor YAP in the cytoplasm (7). 14-3-3 proteins are a family of proteins involved in a multitude of diverse signaling pathways. Interestingly, T. gondii secretes its own 14-3-3 protein (Tg14-3-3) into host cells, which interacts with host 14-3-3 proteins at the parasitophorous vacuole membrane (PVM) and contributes to the hypermotility of T. gondii-infected dendritic cells (59). Although YAP does not appear to localize at the PVM in T. gondii*-*infected endothelial cells, the possibility of cross talk between host and parasite-secreted 14-3-3 proteins in modulating YAP activity may be an interesting avenue for investigation.

The current research provides evidence that T. gondii remodels components of the endothelial cell cytoskeleton and alters endothelial cell barrier function during infection. Because the actin cytoskeleton, through its interactions with binding partners and junction proteins, lies at the heart of mechanotransduction signaling, these findings also provide new insights into host cell biomechanical sensing of intracellular T. gondii infection.

## MATERIALS AND METHODS

### Mammalian and parasite cell culture.

Human umbilical vein endothelial cells (HUVEC) (Lonza, Allendale, NJ) were cultured in endothelial growth medium 2 (EGM-2) with EGM-2 SingQuot supplements and growth factors (Lonza) or in EGM (R&D Systems, Minneapolis, MN). GFP-expressing and tdTomato-expressing type II T. gondii tachyzoites (*Prugniaud* strain) were maintained in human foreskin fibroblasts (HFF) and syringe lysed, and washed immediately before experimentation as previously described (60). Briefly, T. gondii-infected HFF monolayers were scraped with a cell scraper, and the resulting cell suspension was syringe lysed with a 27 1/2 gauge syringe before bringing to a final volume of 15 ml with fresh D-10% medium and spinning at 1,500 rpm for 7 min at room temperature (RT). Next, the parasite pellet was resuspended with 3 ml fresh D-10% before passage through a 0.5-μm filter to remove cellular debris. The filtrate was brought to a final volume of 15 ml with fresh D-10% before spinning at 1,500 rpm for 7 min at RT. Lysed and filtered parasites were resuspended in 1 ml fresh EGM and counted with a hemocytometer. HUVEC were infected at an MOI of 1 or 2. Mock-infected cells were those to which prewarmed fresh medium was added in the place of parasites. All parasite and human cell cultures were routinely tested for *Mycoplasma* contamination and confirmed to be negative.

### HUVEC TEER assays.

A 96-well electrode array comprised of 20 interdigitated electrode fingers with a total area of 3.92 mm^2^ (Applied Biophysics, Troy, NY) was coated with 20 μg/ml fibronectin, and 4 × 10^4^ to 5 × 10^4^ HUVEC were seeded into each well. Immediately after seeding, the array was placed on an ECIS Zθ (Applied Biophysics) and maintained at 37°C and 5% CO_2_. The impedance in each well was measured every 15 min at multiple alternating current (AC) frequencies. HUVEC were provided fresh media at 24 and 48 h after seeding. At 72 h after initial seeding, 0.5 ng/ml IL-1β, syringe-lysed T. gondii tachyzoites at an MOI of 1 or 2, or fresh media were added to the test wells, and the assay was conducted for an additional 24 h. Impedance measured at 4,000 Hz and 64,000 Hz was used to calculate resistance and capacitance, respectively. Resistance was then multiplied by the surface area of the electrical cell-substrate impedance sensing (ECIS) electrodes to calculate transendothelial electrical resistance (TEER) values (61).

### Microfluidics.

Microfluidic experiments were performed using the ibidi pump system (ibidi GmbH, Martinsried, Germany). For shear stress conditions, 2.5 × 10^5^ HUVEC were seeded into ibiTreated μ-slide I 0.4 Luer chambers and cultured under static conditions at 37°C with 5% CO_2_ for 1 to 3 h. The μ-slide was then connected to the ibidi pump system, and HUVEC monolayers were cultured to confluence under 5.5 dyne/cm^2^ of continuous flow for 72 h. After the cells reached confluence, flow was paused, and syringe-lysed tachyzoites or fresh media were added to monolayers and cultured under static conditions for 1 h. The μ-slide was then reconnected to flow and cultured under shear conditions for an additional 17 h.

### Immunofluorescence microscopy.

For static cultures, 2.5 × 10^5^ HUVEC were seeded onto fibronectin-coated coverslips and cultured to confluence at 37°C and 5% CO_2_ for 72 h. Syringe-lysed tachyzoites or fresh media were added, and HUVEC were cultured for an additional 18 h. For shear stress conditions, the μ-slide containing mock-infected or T. gondii-infected HUVEC (described above) was disconnected from the flow pump. Coverslips or μ-slides were then washed with phosphate-buffered saline (PBS), fixed with 4% paraformaldehyde (PFA) (Electron Microscopy Sciences, Hatfield, PA), blocked with PBS containing 5% normal goat serum (NGS) (Southern Biotech, Birmingham, AL), and permeabilized with 0.1% Triton X-100 (Sigma, St. Louis, MO).

HUVEC were stained with antibodies recognizing VE-cadherin (BV9; Biolegend, San Diego, CA), β-catenin (Cell Signaling Technologies, Danvers, MA), YAP (G-6; Santa Cruz Biotech, Dallas, TX), and gm130 (Sigma) and with Hoechst dye (Life Technologies, Carlsbad, CA). Alexa Fluor 488 (AF 488)-conjugated goat anti-mouse IgG, AF 594-conjugated goat anti-mouse IgG, AF 647-conugated goat anti-mouse IgG, and AF 594-conjugated anti-rabbit IgG, or AF 647-conjugated goat anti-rabbit IgG (all from Life Technologies) were used as secondary antibodies. For visualization of F-actin, monolayers were incubated with AF 594-conjugated phalloidin (Life Technologies). Coverslips were mounted using Vectashield with or without DAPI (Vector Laboratories, Burlingame, CA). For shear stress conditions, μ-slides were sealed with ibidi’s mounting medium. Confocal microscopy was performed using a Leica SP-8 microscope with a 63× objective, and data were analyzed using ImageJ software. Prism (GraphPad Software, La Jolla, CA) was used to graph data and perform statistics.

### Quantification of fluorescence microscopy images.

Quantification of cellular structures was performed on confocal images from at least three independent experiments per analysis. Images were analyzed using ImageJ. Discontinuity in VE-cadherin or β-catenin staining was defined by the absence of fluorescence signal at the cell periphery measuring a distance 2.5 μm or greater. To quantify cell numbers and morphology, DAPI-stained nuclei per 63× field of view (FOV) were counted, and only nuclei that were completely visible in the FOV were counted. To measure the maximal length of each cell, the cell boundaries were marked by VE-cadherin staining or mCLING-647 (Synaptic Systems, Goettingen, Germany). Maximal length was determined as the longest distance across the cell spanning the nucleus. These quantifications were done manually in a nonblind manner. To quantify F-actin stress fiber abundance, the percent area of F-actin per FOV was determined in a semiautomated manner: F-actin signal was uniformly thresholded and binarized across samples and converted into a mask. The area covered by F-actin in the 63× FOV was determined. For analysis of F-actin in individual cells, the cell boundaries were marked by VE-cadherin staining or mCLING, and a duplicate image of single cells was generated. F-actin signal was thresholded and binarized before converting into a mask. Cytoplasmic F-actin signal was measured by selecting only signal within cell boundaries. These quantifications were done in a nonblind manner. To quantify planar cell polarity, the position of the Golgi apparatus with respect to the nucleus was determined. A 120° arc was drawn from the center of the nucleus to the cell periphery, in line with the direction of shear flow. Golgi structures fully or at least halfway positioned within this arc were counted as polarized. Golgi apparatus less than halfway positioned within this arc or completely outside the arc were counted as not polarized. Only HUVEC whose entire nucleus was in the FOV were counted.

### Immunoblots.

A total of 2.5 × 10^5^ to 5 × 10^5^ HUVEC were seeded into fibronectin-coated wells of a six-well plate and cultured to confluence at 37°C and 5% CO_2_ for 72 h. Syringe-lysed tachyzoites or fresh media were added to the cells, which were then cultured for an additional 18 h. As a positive control for phospho-LATS1 detection, confluent HUVEC monolayers were treated with 100 ng/ml 12-*O*-tetradecanoylphorbol-13-acetate (TPA) (Sigma) for 18 h. Monolayers were directly lysed in 2× Laemmli buffer supplemented with 10% 2-mercaptoethanol. Collected lysates were boiled for 20 min at 95 to 100°C. Lysates were separated on 10% sodium dodecyl sulfate (SDS)-polyacrylamide gels, transferred to polyvinylidene difluoride (PVDF) membranes, and immunoblotted with mouse anti-VE-cadherin (BV9; BioLegend), rabbit-anti-β-catenin (Cell Signaling Technologies), mouse anti-YAP (G-6; Santa Cruz Biotech), rabbit anti-LATS1 (C66B5, Cell Signaling Technologies), rabbit anti-phospho-LATS1 (Thr1079, D57D3; Cell Signaling Technologies), or mouse anti-glyceraldehyde-3-phosphate dehydrogenase (anti-GAPDH) monoclonal antibodies (MAbs) (FF26A/F9; BioLegend). The membranes were then stained with horseradish peroxidase (HRP)-conjugated anti-mouse or anti-rabbit IgG (Jackson Immuno Research, West Grove, PA), developed using enhanced chemiluminescence (ECL), ECL prime, or SuperSignal (GE Healthcare Life Sciences, Pittsburgh, PA) and detected using a Nikon camera as previously described (62). Densitometry was performed using ImageJ software.

### Transwell assays.

HUVEC were seeded onto fibronectin-coated transwell membranes (pore size, 0.4 μm; exposed area, 1.12 cm^2^; Costar) and cultured to confluence at 37°C and 5% CO_2_ for 72 h. At the time of the assay, 0.5 ng/ml IL-1β, syringe-lysed tachyzoites, or fresh media were added to the apical chamber containing HUVEC, and the cells were cultured for an additional 18 h. In the final 30 min, 40-kDa FITC-dextran (Life Technologies) at 1 mg/ml concentration was added to the apical chamber, and the monolayers were cultured for an additional 30 min. Transwell inserts were removed, and medium from the basolateral chamber was collected and triplicate plated into a 96-well plate (Life Technologies). Fluorescence was measured using a spectrophotometer with 480-nm and 528-nm excitation and emission wavelengths, respectively (Synergy H1 hybrid, BioTek Instruments, Inc., Winooski, VT; SpectraMAX XPS, Molecular Devices, Sunnyvale, CA). The concentrations of FITC-dextran under each condition were determined using a standard curve.

### RNA isolation and library preparation.

Three independent experiments were performed in which HUVEC were mock infected or infected with T. gondii. Total RNA from each set of experimental samples was isolated using the Qiagen RNeasy kit (Qiagen, Valencia, CA). RNA concentration and integrity were determined using an Agilent 2100 bioanalyzer (Agilent Technologies, Santa Clara, CA). One hundred nanograms of total RNA was treated with 1 U of DNase I (New England BioLabs) at 37°C for 10 min and cleaned with RNACleanXP beads (Beckman Coulter). Next, poly(A) mRNA was purified using Illumina’s oligo(dT) beads, followed by fragmentation at 94°C for 8.5 min to yield a median insert length of 155 nucleotides (nt). Libraries were prepared using the TruSeq RNA Library Prep kit v2 according to the manufacturer’s instructions. Each library was prepared with a unique indexed primer for multiplexing. In order to ensure proper sizing, quantitation, and quality prior to sequencing, libraries were analyzed using Agilent High Sensitivity DNA kit. Multiplexed libraries were sequenced in single-end 86-bp reads using the NextSeq 500 platform (Illumina, San Diego, CA).

### RNA-Seq bioinformatic analysis.

Data analysis was performed with the RNA-Seq workflow module of the *systemPiperR* package available on Bioconductor (63). RNA-Seq reads were demultiplexed using bcl2fastq conversion software. Sequence quality was assessed using FastQC function and trimmed using Trim Galore with an average phred score cutoff of 30 and minimum length of 65 bp. Read sequences were trimmed 10 nt at the 5′ end and 2 nt at the 3′ end to avoid poor-quality bases. Trimmed sequences were aligned to the Homo sapiens reference genome (hg38) using TopHat2/Bowtie2 suite with the corresponding annotation file from Ensembl (Homo_sapiens.GRCh38.p10.gtf). Raw expression values in the form of gene-level read counts were generated with the *summarizeOverlaps* function, counting only the reads overlapping exonic regions of genes, and discarding reads mapping to ambiguous regions of exons from overlapping genes. Normalization and statistical analysis of differentially expressed genes (DEGs) were performed using the *edgeR* package, which normalizes reads by the trimmed mean of M-value method. DEGs were defined as those with a fold change of >2 or <0.5 and a Benjamini-Hochberg-controlled false-discovery rate (FDR)-corrected *P* value of <0.05 compared to mock-infected samples. Only protein-coding genes with an average of greater than five reads per kilobase of transcript per million mapped reads (RPKM) were included for further analysis. Heatmaps for gene expression represent the absolute normalized expression (transcripts per million [TPM]); the range of colors is based on scaled and centered TPM values of the entire set of genes (red represents high expression, whereas blue represents low expression).

### Functional enrichment.

Functional enrichment analysis was performed to identify biological pathways, including Gene Ontology (GO) terms, using Metascape (64). Significant functional enrichment terms were defined as those with a FDR-corrected *P* value of <0.05.

### qPCR.

Total RNA was harvested from 0.5 × 10^6^ to 1 × 10^6^ mock-infected or T. gondii-infected HUVEC using the QiaShredder homogenizer and RNeasy kit (Qiagen). cDNA was synthesized as described previously (60) and added to iQ SYBR green SuperMix (Bio-Rad Laboratories, Hercules, CA) using the following primers: CTGF (sense) 5′-ACCGACTGGAAGACACGTTTG-3′, CTGF (antisense) 5′-CCAGGTCAGCTTCGCAAGG-3′, CYR61 (sense) 5′-GCAAGGAGCTGGGATTCGAT-3′, CYR61 (antisense) 5′-GGCTCCATTCCAAAAACAGGG-3′, ANKRD1 (sense) 5′-CACTTCTAGCCCACCCTGTGA-3′, ANKRD1 (antisense) 5′-CCACAGGTTCCGTAATGATTT-3′, GAPDH (sense) 5′-GAAGGTGAAGGTCGGAGT-3′, and GAPDH (antisense) 5′-GAAGATGGTGATGGGATTTC-3′. The data from the qPCR were analyzed using the ΔΔ*C_T_* (comparative threshold) method (65). The values obtained for expression were normalized to those of GAPDH, and the data are expressed as a ratio of mRNA levels. Error bars represent the standard errors of the means (SEM) from three independent experiments. No signals were detected when water or samples generated without reverse transcriptase were used as the template.

### Statistics.

Statistical analysis was performed using Prism software. Student’s *t* test was used for comparisons between mock-infected and T. gondii-infected samples. Two-way analysis of variance (ANOVA) with a Bonferroni posthoc test was used for TEER data. One-way ANOVA with a Tukey posthoc test was used for all experiments with multiple comparisons.
