# Supplementary figures and images for: Toxoplasma gondii Dysregulates Barrier Function and Mechanotransduction Signaling in Human Endothelial Cells
Source: mSphere. 2020 Jan 29;5(1):e00550-19. doi: 10.1128/mSphere.00550-19 (PMC6992369; doi:10.1128/mSphere.00550-19)

Supplemental Figure 1

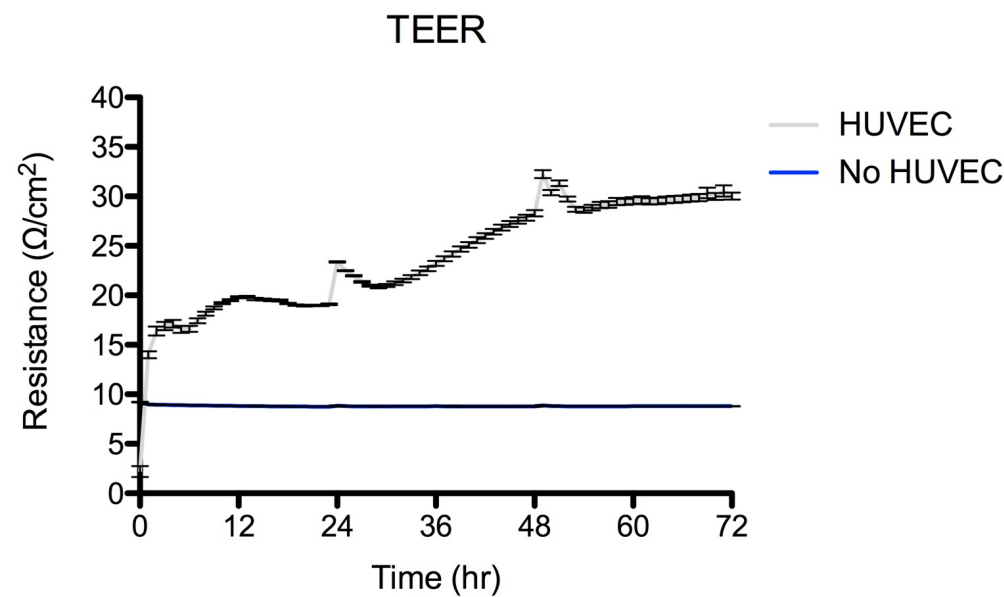

Supplement: FIG S1 [file mSphere.00550-19-sf001.pdf]
